# Association between previous schistosome infection and incident hyperuricemia: A prospective cohort study in China

**DOI:** 10.1371/journal.pone.0212702

**Published:** 2019-02-21

**Authors:** Guangli Wang, Yang Jing, Hui Zhou, Yi Ding, Jie Wang, Jing Qiu, Haiyong Hua, Chen Dong

**Affiliations:** 1 Department of Epidemiology and Statistics, School of Public Health, Jiangsu Key Laboratory and Translational Medicine for Geriatric Disease, Medical College of Soochow University, Suzhou City, Jiangsu Province, China; 2 Suzhou Industrial Park Centers for Disease Control and Prevention, Suzhou City, Jiangsu Province, China; 3 Jiangsu Institute of Parasitic Diseases, Wuxi City, Jiangsu Province, China; Penn State University, UNITED STATES

## Abstract

**Background:**

More than 11 million people were estimated to be infected by Schistosoma japonicum in China before the 1950s. However, seldom studies have been conducted to evaluate the longitudinal effects of previous schistosome infection (PSI). We aimed to investigate the association between PSI and hyperuricemia in China.

**Methods:**

From February 2013 to October 2013, 3,517 Chinese subjects (908 persons with PSI and 2,609 persons without PSI) were recruited from a prospective cohort study of “135”. After two years, 113 and 462 participants had developed hyperuricemia in the persons with and without PSI, respectively. Multivariable logistic models were used to estimate Relative Ratios (RR) and 95% confidence intervals (CIs) for hyperuricemia.

**Results:**

The PSI participants had a decreased risk of hyperuricemia compared with those without PSI [adjusted RR (95%CI): 0.73 (0.55, 0.97)]. Within the PSI group, higher level of fasting plasma glucose was significantly associated with the reduced incidence of hyperuricemia in PSI population (RR: 0.40, 95% CI: 0.26–0.63). For females, hypertension, increased levels of serum creatinine and triglycerides were the risk factors for incident hyperuricemia in the PSI group.

**Conclusions:**

Our results suggest that PSI is significantly associated with the lower incidence of hyperuricemia. Moreover, elevated fasting plasma glucose might prevent the onset of hyperuricemia in PSI population.

## Introduction

Schistosomiasis japonica, caused by human blood fluke *Schistosoma japonicum*, is an important public health concern and affects more than 260 million people worldwide, especially in tropical and subtropical regions. In China, more than 11 million people were threatened by the disease before 1950 [1]. The implementation of National Middle- and Long-term Plan of Schistosomiasis Prevention and Control has resulted in remarkable progress, with an overall downward trend in the prevalence and intensity of *S*. *japonicum* infection [2]. Schistosomiasis is not only a helminth infection but also an immunological disease [3, 4]. Animal studies have shown that schistosome antigens may affect the metabolic profiles and induce a strong anti-inflammatory response [5, 6].

Serum uric acid (sUA) is an end product of purine metabolism and is related to the purine bases of nucleic acids [7]. In physiological conditions, the levels of sUA are maintained by the balance between uric acid production and excretion. In general, hyperuricemia occurs when purine intake through food or the formation of endogenous purine due to cell turnover increases the concentrations of sUA or when the amount of uric acid excreted through the kidney diminishes. Hyperuricemia is usually recognized as the principal cause of inflammatory arthritis [8]. However, an increasing number of studies conducted in the past decade have demonstrated that hyperuricemia is an independent risk factor for metabolic and cardiorenovascular diseases, including hypertension, metabolic syndrome, nonalcoholic fatty liver disease and type 2 diabetes (T2DM) [9–11].

Although the clinical characteristics and the outcomes of acute or chronic *S*. *japonicum* infection have been described in detail before, the long-term effect of previous schistosome infection (PSI) on the health condition of the affected individual remains unclear. Several recent epidemiological studies from China have reported a significant association of PSI with decreased risks for T2DM, metabolic syndrome and cardiovascular diseases [6, 12–14]. Considering that hyperuricemia is an established risk factor for metabolic syndrome and cardiorenovascular diseases, we conducted the prospective cohort study to evaluate whether PSI may reduce the risk of hyperuricemia. Furthermore, the potential factors associated with the incidence of hyperuricemia in population with PSI were analyzed herein.

## Materials and methods

### Study participants

**Fig 1:**
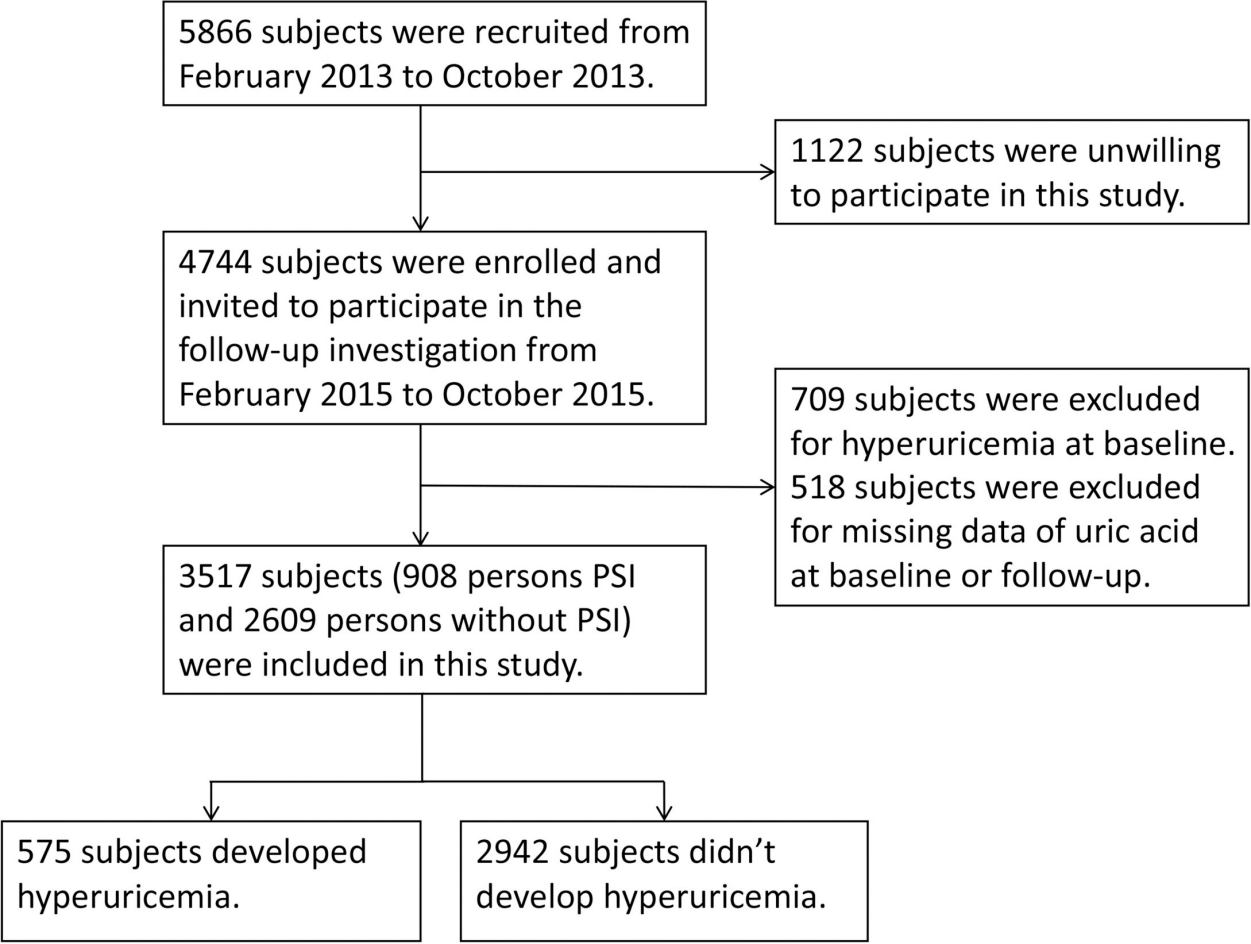
Flow diagram of study.

The study participants were recruited from a prospective cohort study of “135”[15], which was established to analyze the prevention strategy for chronic diseases in Soochow, China. The design of the “135” study has been previously published[15]. Briefly, two-stage sampling method was used for the recruitment of the participants. First, three communities were randomly selected from Soochow. Then, a total of 5,866 unrelated participants in three communities with aged from 35 to 70 years was enrolled from between February 2013 and October 2013. Among them, 4,744 individuals were invited to participate in the 2-year follow-up between February 2015 and October 2015. After the exclusion of 709 subjects with hyperuricemia and 518 subjects with missing data of uric acid at baseline, 3,517 subjects (908 persons with PSI and 2,609 subjects without PSI) who completed a large-scale physical examination were included in the present study (Fig 1).

This work was approved by the ethical committee of Soochow University and was conducted in accordance with the ethical standards of the responsible committees for human experimentation and with the Helsinki Declaration of 1975. All of participants were asked to provide written informed consent before data collection.

### Baseline measurements

The demographic information, lifestyle risk factors (smoking and drinking), history of disease (hypertension, T2DM and chronic kidney disease) of participants were collected by trained interviewer. Body weight and height were measured according to standard methods. Three seated blood pressure (BP) measurements were obtained by trained staff after at least five minutes of quiet rest.

Blood samples were drawn from each participant after fasting at least 10 h. Fasting plasma glucose (FPG), total cholesterol, LDL cholesterol, HDL cholesterol and triglycerides were measured using an ADVIA1650 Automatic Analyzer in the same laboratory. Serum uric acid (sUA) was measured with the Fossati enzymatic reaction using uricase as a Trinder-like endpoint (ADVIA1650 Autoanalyzer). Serum creatinine was measured using an enzymatic method on an Ortho Vitros 950 and standardized to isotope-dilution mass spectrometry traceable values.

### Definitions

In the present study, PSI was defined by self-reported schistosome infection and was validated by Liver B ultrasound, hypertension was defined as SBP ≥ 140 mmHg and/or DBP ≥ 90 mmHg, or self-reported use of antihypertensive medication. Diabetes mellitus was defined as FPG ≥ 7.0mmol/L, or self-reported use of anti-diabetes mellitus medication. The outcome of the present study was hyperuricemia, which was assessed as an individual with > 416 μmol/L of sUA level for men and > 357 μmol/L for women.

### Statistical analysis

Descriptive statistics are presented as mean ± standard deviation for continuous variables (median [interquartile range] for skewed variables) and as number (percentage) for categorical variables. Comparisons between different groups were performed using Student’s t or ANOVA tests for continuous variables with normal distribution, and Wilcoxon rank-sum tests or Kruskal-Wallis H tests for variables with skewed distribution, respectively. Chi-square tests were used to compare categorical variables. Logistic regression analysis was used to estimate the relative ratio (RR) (95% CI) of the outcome of hyperuricemia in participants with PSI compared to that in subjects without PSI. Four statistical models were used in this study. The preliminary model was crude and considered only PSI exposure, whereas Model 1 was adjusted for age and gender, Model 2 was adjusted for the variables in Model 1 and for BMI, smoking status and drinking status, and Model 3 was adjusted for the variables in Model 1 and 2 in addition to hypertension status, FPG, total cholesterol, triglycerides and serum creatinine. To explore the potential factors associated with hyperuricemia in the PSI population, a forest plot was used to depict the results of RR and 95% CI after adjusted for other confounding factors. All statistical analyses were conducted using SAS statistical software (version 9.4, Cary, NC, USA) and two-tailed p < 0.05 was considered as statistically significant.

## Results

### Baseline characteristics of study population

Basic characteristics of the study population with and without PSI are summarized in Table 1. Among 3517 participants included in the present study, 908 (27.87%) subjects had been infected by schistosome in the past and 45.81% of PSI participants were males. The median age of the PSI group was 60 (56–64) years, which was significantly older than those participants without PSI. But the median BMI of PSI population was 23.12 (21.22–24.89) Kg/m^2^, which was significantly lower than those without PSI (P<0.0001). In addition, the results showed that the persons with PSI had lower level of triglycerides and higher level of HDL cholesterol compared to non-PSI population.

**Table 1:** Characteristics of the study population with and without PSI.

Items^a^	Total	With PSI	Without PSI	P Value
N	3517	908 (27.87)	2609 (72.13)	
Age (years)	51 (46–60)	60 (56–64)	49 (45–57)	<0.0001
Male (%)	1432 (40.72)	416 (45.81)	1016 (38.94)	0.0003
Occupations				<0.0001
Mental workers	656(18.65)	85(9.36)	571(21.89)	
Manual workers	1694(48.17)	474(52.2)	1220(46.76)	
Retirees	518(14.73)	170(18.72)	348(13.34)	
Others	649(18.45)	179(19.71)	470(18.01)	
Smoking status (%)	861 (24.48)	242 (26.65)	619 (23.73)	0.0773
Drinking status (%)	644 (18.31)	157 (17.29)	487 (18.67)	0.3560
BMI (Kg/m^2^)	23.42 (21.53–25.43)	23.12 (21.22–24.89)	23.45 (21.63–25.61)	<0.0001
Hypertension (%)	898 (25.53)	251 (27.64)	647 (24.80)	0.0905
FPG (mmol/L)	5.66 (5.19–6.11)	5.69 (5.35–5.89)	5.61 (5.16–6.27)	0.0678
Serum Creatinine (umol/L)	69 (59–82)	68 (59–80)	69 (58–82)	0.4070
sUA (umol/L)	290 (246–336)	293 (252–337)	289 (245–336)	0.0760
Total cholesterol (mmol/L)	4.73 (4.16–5.35)	4.71 (4.18–5.38)	4.74 (4.16–5.33)	0.8069
Triglycerides (mmol/L)	1.18 (0.86–1.70)	1.02 (0.79–1.44)	1.26 (0.89–1.79)	<0.0001
HDL cholesterol (mmol/L)	1.32 (1.08–1.56)	1.36 (1.14–1.60)	1.30 (1.06–1.54)	<0.0001
LDL cholesterol (mmol/L)	2.79 (2.34–3.28)	2.76 (2.30–3.23)	2.80 (2.35–3.29)	0.1417

^a^Data are expressed as median (25th-75th percentile) or n (%).BMI, Body Mass Index; FPG, Fasing Plasma Glucose; sUA, serum Uric Acid; PSI, Previous Schistosome Infection.

In total, 575 participants were diagnosed with hyperuricemia at the end of study. As the results shown in Table 2, the incidence of hyperuricemia was higher among males, and among the participants with hypertension, habits of smoking and drinking, and manual labor. Moreover, those who developed hyperuricemia were more likely to have higher levels of BMI, total cholesterol, triglycerides and HDL cholesterol, but to have lower levels of LDL cholesterol and FPG compared to the participants without hyperuricemia.

**Table 2:** Characteristics of the study population with and without hyperuricemia during follow-up.

Items^a^	Total	Hyperuricemia	Non-hyperuricemia	P Value
N	3517	575(16.35)	2942(83.65)	
Age (years)	51(46–60)	54(47–61)	51(46–60)	0.0022
Male (%)	1432(40.72)	266(46.26)	1166(39.63)	0.0031
Occupations				<0.0001
Mental workers	656(18.65)	70(12.17)	586(19.92)	
Manual workers	1694(48.17)	307(53.39)	1387(47.14)	
Retirees	518(14.73)	62(10.78)	456(15.5)	
Others	649(18.45)	136(23.65)	513(17.44)	
Smoking status (%)	898(25.53)	168(29.22)	693(23.56)	0.0039
Drinking status (%)	5.66(5.19–6.11)	134(23.30)	510(17.34)	0.0007
BMI (Kg/m^2^)	23.42(21.53–25.43)	24.45(22.50–26.4)	23.19(21.37–25.24)	<0.0001
Hypertension (%)	644(18.31)	239(41.57)	659(22.40)	<0.0001
FPG (mmol/L)	861(24.48)	5.21(4.96–5.62)	5.73(5.31–6.20)	<0.0001
Serum Creatinine (umol/L)	69(59–82)	72(60–85)	68(58–81)	<0.0001
sUA (umol/L)	290 (246–336)	340 (308–369)	280 (239–324)	<0.0001
Total cholesterol (mmol/L)	4.73(4.16–5.35)	4.98(4.40–5.60)	4.67(4.12–5.28)	<0.0001
Triglycerides (mmol/L)	1.18(0.86–1.70)	1.41(0.97–2.03)	1.14(0.84–1.64)	<0.0001
HDL cholesterol (mmol/L)	1.32(1.08–1.56)	1.47(1.27–1.63)	1.29(1.06–1.53)	<0.0001
LDL cholesterol (mmol/L)	2.79(2.34–3.28)	2.70(2.30–3.16)	2.80(2.35–3.29)	0.0060

^a^Data are expressed as median (25th-75th percentile) or n (%). BMI, Body Mass Index; FPG, Fasing Plasma Glucose; sUA, serum Uric Acid; PSI, Previous Schistosome Infection.

### Associations between PSI and incident hyperuricemia

We applied logistic regression models to assess the incidence of hyperuricemia of PSI and non-PSI. As the results shown in Table 3, compared with participants without PSI, the RR (95% CI) of 2-year incidence of hyperuricemia for those with PSI was 0.66 (0.53–0.82), 0.49 (0.39–0.63) and 0.57(0.44–0.74) in unadjusted analysis, model 1 and model 2, respectively. After adjusting for age, gender, smoking status, drinking status, BMI, hypertension status, total cholesterol, triglycerides, FPG, serum creatinine and occupations at baseline in model 3, PSI was still associated with a decreased risk of incident hyperuricemia (RR: 0.73, 95% CI: 0.55–0.97). Therefore, our results suggested that PSI was the independent factor of decreased risk of incident hyperuricemia in Chinese adults.

**Table 3:** Analysis on the associations between PSI and hyperuricemia.

Items^b^	Without PSI^a^	With PSI^a^	P Value
Number of case (%)	462(17.71)	113(12.44)	
Crude	1.00(ref)	0.66(0.53–0.82)	0.0002
Model1	1.00(ref)	0.49(0.39–0.63)	<0.0001
Model2	1.00(ref)	0.57(0.44–0.74)	<0.0001
Model3	1.00(ref)	0.73(0.55–0.97)	0.0289

^a^PSI, Previous Schistosome Infection

^b^Crude model was unadjusted, Model 1 was adjusted for age and gender, Model 2 was adjusted for model 1 variables and smoking status, drinking status, body mass index, Model 3 was adjusted for model 2 variables and hypertension status, serum creatinine, triglycerides, total cholesterol, fasting plasma glucose and occupation.

### Risk factor for incident hyperuricemia in the PSI group

Next, we analyzed the factors associated with the risk of hyperuricemia in PSI population. Among 908 PSI participants included in the present study, 113 persons developed hyperuricemia. Table 4 displayed the baseline characteristics of PSI individuals with hyperuricemia compared to those without hyperuricemia. The results showed that PSI participants who developed hyperuricemia were more likely to have higher levels of serum creatinine, total cholesterol, triglycerides and HDL cholesterol, but to have lower level of FPG. Moreover, the incidence of hyperuricemia was significantly higher among the participants with hypertension.

**Table 4:** Characteristics of PSI participants with or without hyperuricemia.

Items^a^	Hyperuricemia	No hyperuricemia	P Value
N	113(12.44)	795(87.56)	
Age (years)	62(57–69)	60(56–64)	0.0043
Gender (% male) Smoking status(% of risk)	55(48.67) 34(30.09)	361(45.41) 208(26.16)	0.5147 0.3772
Occupations			0.1581
Mental workers	14(12.39)	71(8.93)	
Manual workers	63(55.75)	411(51.7)	
Retirees	13(11.5)	157(19.75)	
Others	23(20.35)	156(19.62)	
Drinking status(% of risk)	26(23.01)	131(16.48)	0.0858
BMI (Kg/m^2^)	24.10(21.73–25.81)	23.01(21.08–24.73)	0.0027
Hypertension (%)	50(42.74)	251(25.56)	<0.0001
FPG (mmol/L)	5.30(5.00–5.72)	5.71(5.42–5.89)	<0.0001
Serum creatinine (umol/L)	72(66–84)	67(58–79)	<0.0001
sUA (umol/L)	343 (312–361)	285 (246–329)	<0.0001
Total cholesterol (mmol/L)	4.87(4.29–5.60)	4.67(4.16–5.35)	0.0247
Triglycerides (mmol/L)	1.21(0.87–1.80)	1.01(0.78–1.37)	0.0002
HDL cholesterol (mmol/L)	1.44(1.24–1.64)	1.36(1.12–1.59)	0.0376
LDL cholesterol (mmol/L)	2.75(2.30–3.23)	2.76(2.31–3.23)	0.8694

^a^Data are expressed as median (25th-75th percentile) or n (%). BMI, Body Mass Index; FPG, Fasing Plasma Glucose; sUA, serum Uric Acid; PSI, Previous Schistosome Infection.

**Fig 2:**
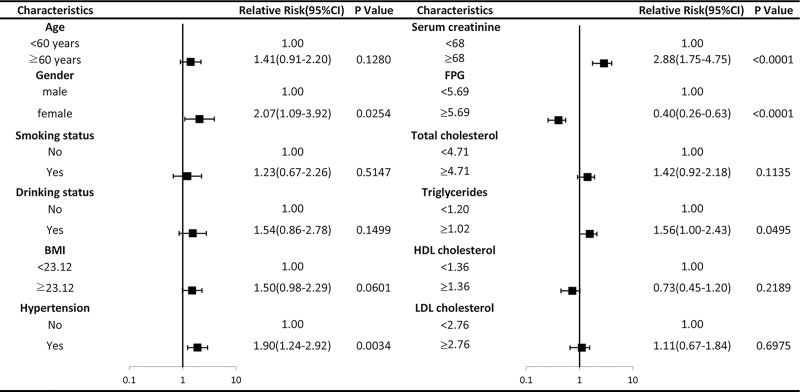
Forest plot of characteristics on relative risk and 95%CI of hyperuricemia in PSI population.

As the results shown in Fig 2, females had an increased risk of hyperuricemia compared to those in males (RR: 2.07, 95% CI: 1.09–3.92). Additionally, hypertension, increased levels of triglycerides and serum creatinine were positively associated with higher incidence of hyperuricemia. However, our present results suggested that the higher level of FPG was significantly associated with the decreased risk of hyperuricemia in PSI population (RR: 0.40, 95% CI: 0.26–0.63). The adjusted RRs with 95% CI of the present analyses were listed in Fig 2.

## Discussion

To our knowledge, this is the first prospective study to investigate the associations of PSI with the incidence of hyperuricemia in China. The results revealed a significant association between PSI and a decreased risk of the incidence of hyperuricemia. Moreover, increased level of FPG is a protective factor, while females, hypertension, increased levels of BMI, serum creatinine and triglycerides were identified as the risk factors for incident hyperuricemia in the PSI population. Previously, several studies have suggested that PSI could improve blood lipid metabolism and reduce the risks of diabetes, metabolic syndrome and cardiovascular diseases [6, 12–14], which may partially support the findings of our study.

It is well known that increased inflammation reaction and immune response involved in the development and progression of hyperuricemia. A cross-sectional study from Poland showed that inflammatory markers, including MCP-1 and hs-CRP in hyperuricemia persons were much higher than those in non-hyperuricemia population[16]. In addition, increased sUA levels were shown to have pro-inflammatory effects, such as activating the transcription factor nuclear factor κ-B, stimulating the renin-angiotensin system and inhibiting neuronal nitric oxide synthase [17–20]. Although the exact negative regulatory mechanisms have not yet been elucidated comletely, the evidence from experimental study has demonstrated that schistosome-induced pulmonary B cells inhibit allergic airway inflammation and display a reduced Th2-driving function, indicating that chronic infections with the helminth *S*. *mansoni* might down-regulate the immune system and then reduce the risk of the incidence of hyperuricemia in the PSI population [21]. In addition, increased levels of inflammatory markers such as hs-CRP were shown to be significantly associated with the development of hyperuricemia. Therefore, the longitudinal effect of PSI on the host immune function and its association with hyperuricemia should be investigated in the future.

Similar to the results of previous studies, our results showed that individuals with increased levels of BMI, serum creatinine, total cholesterol and triglycerides have a higher risk for the development of hyperuricemia. However, we observed that participants with elevated FPG levels are less susceptible to be with hyperuricemia. Furthermore, the baseline levels of serum FPG in PSI persons without hyperuricemia also significantly higher than those in the subjects with PSI who developed hyperuricemia. Although several studies have reported that sUA concentrations were positively related with the FPG level [10, 22], seldom studies were performed to explore the effects of serum FPG on the incidence of hyperuricemia. Therefore, it is difficult to provide an accurate explanation of the mechanisms underlying our present observations. In general, both uric acid and glucose are totally filtered in the renal glomerular and almost completely reabsorbed in the proximal tubular [23–26]. Therefore, we hypothesized that increased glucose levels might competitively inhibit uric acid reabsorption and enhances its excretion at the same anatomic position, given a normal renal function.

The major limitations of our present primary study are the small sample size and the short follow-up time. Additionally, we did not measure the levels of inflammatory and immunological markers because of the limited volume of blood samples. Therefore, we are not able to further investigate the immune conditions in individuals with PSI and their effects on the incidence of hyperuricemia.

In conclusion, the results of our study suggest that PSI is significantly associated with a decreased incidence of hyperuricemia. Furthermore, increased serum FPG levels may reduce the risk of hyperuricemia in the PSI population. A longitudinal follow-up study with a large sample size would be necessary to further address the protective ability induced by PSI.

## Supporting information

S1 Table The original information of studied population for baseline analysis. (XLSX)

S2 Table The original information of PSI population for longitudinal analysis. (XLSX)
